# Contrasting Patterns of Transposable Element Insertions in *Drosophila* Heat-Shock Promoters

**DOI:** 10.1371/journal.pone.0008486

**Published:** 2009-12-29

**Authors:** Robert A. Haney, Martin E. Feder

**Affiliations:** Department of Organismal Biology and Anatomy, University of Chicago, Chicago, Illinois, United States of America; Indiana University, United States of America

## Abstract

The proximal promoter regions of heat-shock genes harbor a remarkable number of *P* transposable element (TE) insertions relative to both positive and negative control proximal promoter regions in natural populations of *Drosophila melanogaster*. We have screened the sequenced genomes of 12 species of *Drosophila* to test whether this pattern is unique to these populations. In the 12 species' genomes, transposable element insertions are no more abundant in promoter regions of single-copy heat-shock genes than in promoters with similar or dissimilar architecture. Also, insertions appear randomly distributed across the promoter region, whereas insertions clustered near the transcription start site in promoters of single-copy heat-shock genes in *D. melanogaster* natural populations. *Hsp70* promoters exhibit more TE insertions per promoter than all other genesets in the 12 species, similarly to in natural populations of *D. melanogaster*. Insertions in the *Hsp70* promoter region, however, cluster away from the transcription start site in the 12 species, but near it in natural populations of *D. melanogaster*. These results suggest that *D. melanogaster* heat-shock promoters are unique in terms of their interaction with transposable elements, and confirm that *Hsp70* promoters are distinctive in TE insertions across *Drosophila*.

## Introduction

The massive accumulation of comparative genomic data due to recent large-scale sequencing projects [1] can help test whether a putatively general pattern in a species is unique or is recurrent in evolution. Here we undertake such a test upon the remarkable abundance of a DNA transposon, the *P* element, in the proximal promoter regions of heat-shock genes in natural populations of the model organism *Drosophila melanogaster* [2]. Previously we have ascribed this situation to the intersection of three processes. First, the promoters of heat-shock genes are specialized for rapid and massive transcription when induced by heat and other stresses. Specializations include a lack of nucleosomes and constitutively decondensed chromatin [3]. Second, some mobile genetic elements, such as the *D. melanogaster P* transposable element (TE), have insertion site preferences related to the physical structure of candidate insertion sites rather than to a specific nucleotide sequence in the host DNA [4]. Importantly, when the insertion site preference corresponds to a physical feature of a class of genes, as in this case, the entire class should be preferentially targeted by the transposable element. In heat-shock promoters, the constitutive decondensation of the chromatin and nucleosome-free regions correspond to the preference of *P* elements for such DNA [5]. Finally, once inserted in the germ line, such TEs may persist due to genetic drift or positive selection [6]–[13] or decay and be lost due to unconstrained degeneration and/or negative selection.

In the specific case of *P* elements in heat-shock promoter regions, the ultimate products of these genes are proteins that are either beneficial or deleterious according to cellular context [14]. Specifically, heat-shock proteins act as beneficial molecular chaperones in the stress response, but at high levels are deleterious in the absence of stress. Inasmuch as insertional mutagenesis of *P* elements typically reduces gene expression, both in general and for heat-shock promoter insertions, conditions favoring low levels of heat-shock proteins ought to select for the preservation of *P* element insertions in these genes. Indeed, the population frequencies of many of the *P* element bearing heat-shock alleles is consistent with positive selection [2], [15].

In principle, this intersection of processes ought to be general and demonstrable outside the case of *D. melanogaster P* elements whenever the underlying conditions obtain. Many aspects, including abundant and active transposable elements, “physical” insertion site preferences, and classes of genes with distinctive promoter architectures, are general. The insertional mutagenesis of the heat-shock genes in *D. melanogaster*, moreover, is a function of the physical disruption of the promoters by inserted DNA, as both non-*P* elements and random DNA of equivalent length have identical impacts on gene expression [16]. Indeed, the only oddities in *D. melanogaster* are that the *P* element has invaded the genome only recently, and that this species can be distinctively prone to heat stress due to its life cycle [17]. These oddities aside, an excess abundance of TEs in heat-shock promoters ought to be detectable across other species if the intersection of processes we hypothesize is general.

The recent sequencing of 11 additional genomes of *Drosophila* species provides a remarkable opportunity to test this expectation. In this study, we extend the work of [2] to enumerate TE insertions specifically into promoter regions across species of *Drosophila* by taking advantage of 12 species genome data together with experimental data on promoter location in *D. melanogaster*. First, we test whether the frequency of TE insertions varies between heat-shock gene promoters and promoters of genes that should have similar or divergent promoter architecture across *Drosophila* species. Second, we examine whether TE insertions in promoters are strongly biased towards *P* elements and occur only in *P*- bearing species, as in *D. melanogaster* [2]. We also investigate characteristics of TE insertions that might be informative as to the forces involved in molding their distribution within promoter regions. We expect that differences in characteristics of TEs, such as length or distance from the transcription start site (TSS), or sequence conservation among shared elements, may be due to differences in the strength and/or direction of selection on TEs between genesets across species, and between heat-shock genes in *D. melanogaster* natural populations and those in the 12 species genomes.

## Results

### Analysis and Distribution of TE Insertions

**Figure 1:**
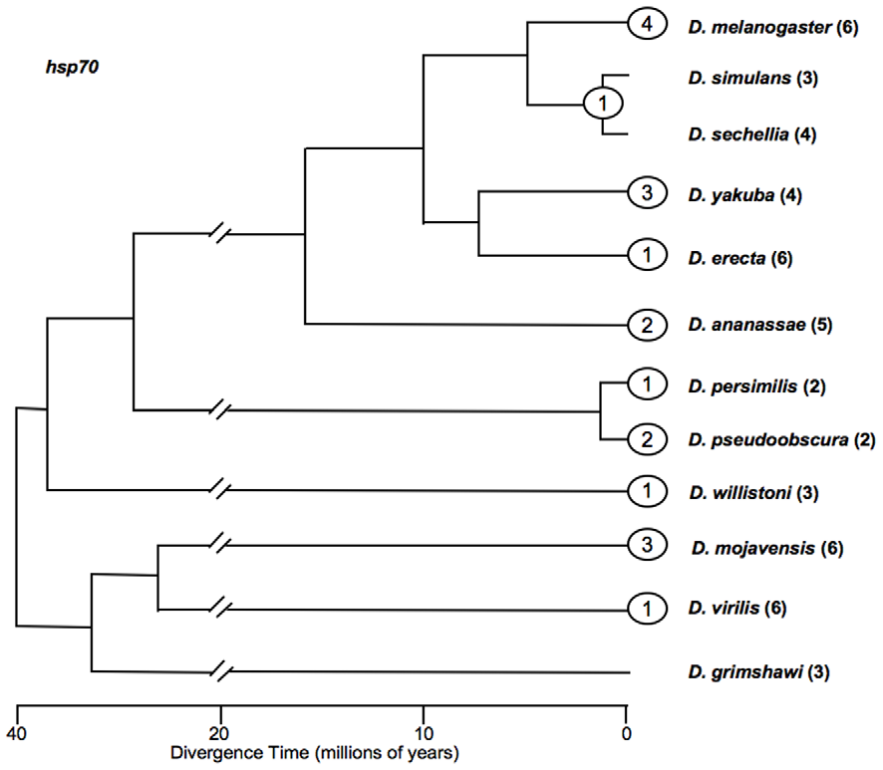
Phylogenetic distribution of transposable element insertions into promoter regions of *Hsp70* in the 12 species of *Drosophila* that have undergone whole-genome sequencing. Each numbered oval represents transposable element insertions. The position of the oval corresponds to the species, species pair, or species group in which the insertion was detected. Ovals at tips are insertions restricted to single species, while ovals at nodes are found in all species emanating from that node. The horizontal scale indicates the time at which the species are presumed to have diverged [59] but is otherwise not pertinent to the time of the TE insertions. Double slashes represent a compression of the time scale at the base of the tree. Numbers following species names in parentheses indicate the number of copies of *Hsp70* per species.

*Hsp70* is uniformly a multi-copy gene in the genus *Drosophila*, with 50 proximal promoter regions (arbitrarily defined as 1000 bp immediately upstream of the TSS) in total computationally identified for the 12 species (Figure 1). In some cases these promoters overlap, thus reducing the total promoter sequence into which TEs could insert to the equivalent of 46 promoters. BLAST analysis of these regions identifies 6 insertions into a single copy within a species, 12 insertions common to >1 copy within a species, and 1 insertion common to 4 paralogs of two species (*D. simulans* and *D. sechellia*). In each common insertion, a given TE is shared and at approximately the same distance from the TSS. By parsimony, the common insertions represent insertion events that antedate gene duplication/conversion and species divergence, respectively.

**Figure 2:**
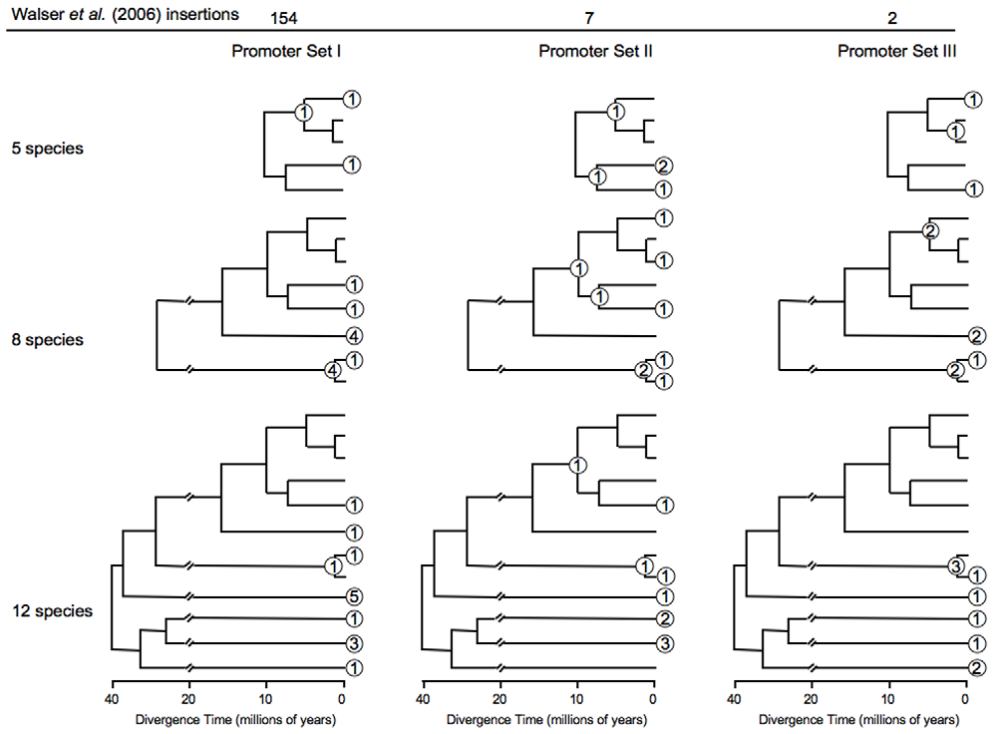
Phylogenetic distribution of transposable element insertions into proximal promoter regions of single-copy genesets in the 12 species of *Drosophila* that have undergone whole-genome sequencing. Species labels are omitted due to space considerations, but are as in Figure 1. Each numbered oval represents transposable element insertions. The position of the oval corresponds to the species, species pair, or species group in which the insertion was detected. Ovals at tips are insertions restricted to single species, while ovals at nodes are found in all species emanating from that node. Insertions are represented at each level of species sampling, and all genes screened at a given level were for the same number of species. The horizontal scale indicates the time at which the species are presumed to have diverged [59] but is otherwise not pertinent to the time of the TE insertions. Double slashes represent a compression of the time scale at the base of the tree.

**Figure 3:**
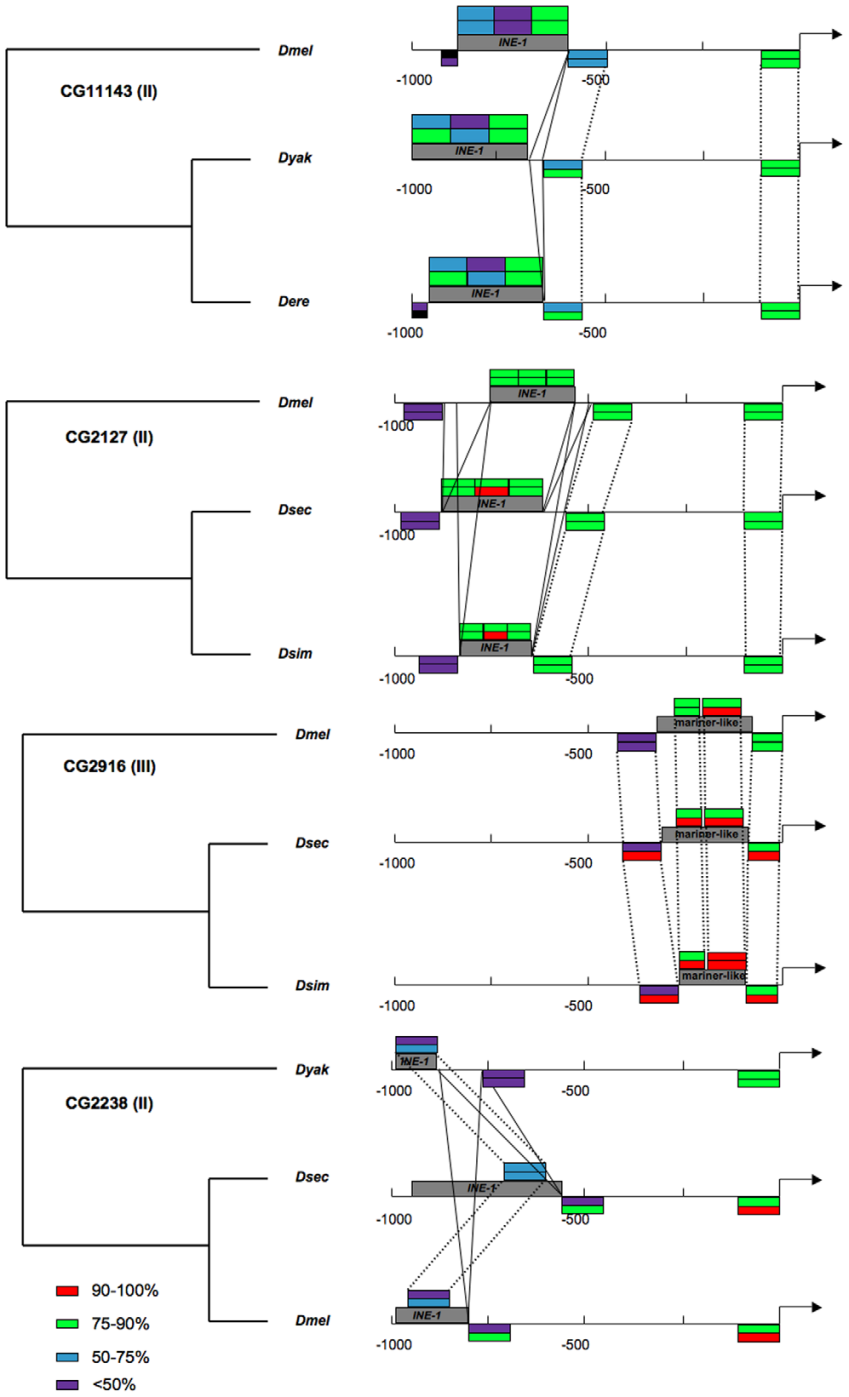
Representative subset of TE insertions in promoter regions shared among 3 species. Sequence conservation of TE and TSS flanking regions is indicated as % pairwise divergence based on Tamura-Nei distance calculated over a 100bp interval of CLUSTAL alignment. Pairwise sequence divergence of TE is based on three equal sized sections of CLUSTAL alignment for CG11143 and CG2127, where elements are of similar size. Solid lines indicate larger indels and dashed lines connect homologous sequence blocks. In CG2916 and CG2238, TE conservation is calculated over conserved blocks corresponding to the smallest element size amongst the three species. The synteny among elements and flanking sequence suggests that these elements are the product of singular insertion events prior to the divergence of the daughter species in which they are currently detectable.

Multi-copy genes such as *Hsp70* present a distinctive target to transposable element insertion and therefore are not readily comparable to single-copy genes. As stated, single-copy genes whose promoters were to be compared were chosen a priori according to characteristics hypothesized to affect the frequency of TE insertions. Of the chosen genes, 36 conserved promoters were recognizable in all 12 species, 45 in only 8 species, and 36 in only 5 species, with equal numbers in each of the 3 sets of genes to be compared (Figure 2). This analysis also revealed that some genes thought to be single-copy have paralogs (based on coding sequence) in some species, of which 8 paralogs in 7 genes had a sufficiently conserved TSS to be included in the analysis (Table S1). BLAST analysis of the 980 individual promoter regions from the 117 distinct TSS of these genes recovered 71 TE insertions (Table S2). 33, 27, and 11 insertions were in the promoters conserved in the 12, 8, and 5 species, respectively. Of these TEs, 49 are present in only a single species (Figure 2). In every other case, the insertion site itself and a major portion of the inserted TE are similar in all species in which the TE appears (see Figure 3 for examples). Where the distance between the insertion site and the TSS is dissimilar in two species putatively sharing a TE, the dissimilarity often corresponds closely to the size of one or more indels. By parsimony, these TEs in >1 species thus represent insertion events that antedate species divergence rather than independent insertion events into multiple species. Only the most recently diverged species (*D. melanogaster* subgroup species/*D. persimilis* and *D. pseudoobscura*) shared insertions. Each of the more widely shared elements appears degenerate, varying extensively among species in both sequence and length (Table S2; Figure 3).

Our goal is to compare numbers of TEs inserted in 3 sets of proximal promoter regions (hereafter “promoter sets”) presenting insertion targets of equal size but hypothesized to differ in susceptibility to gain and/or loss of TEs. Although the 3 promoter sets offer equal numbers of promoters and we have screened 1000 bp upstream of the TSSs in every case, their overall target sizes differ in several ways: (a) <1000 bp may separate TSSs, (b) proximal promoter sequences upstream of two TSSs may overlap, (c) inserted TEs occupy part of the 1000 bp screened, and (d) a 1000 bp window containing a TE is inherited. In each case, equating the target size to 1000 bp may overestimate it. We have corrected target size for (a) in every case. We compare the 3 promoter sets (and *Hsp70*) by estimating aggregate target size in several ways: conservatively [correcting for (b), (c), and (d)], semi-conservatively [correcting for (b) and (c) only], and liberally [correcting for (b) only].

The pattern for natural populations of *D. melanogaster* [2], i.e., vastly more promoters with TEs in that study's promoter sets I (n = 154) than in its promoter sets II (n = 7) and III (n = 2), was not evident in the similarly-defined but non-identical promoter sets of the present study regardless of how the aggregate target size is corrected. Promoter set I of the present study comprises 328–337 kB of promoter sequence depending on whether the correction is conservative, semi-conservative, or liberal (Table S3). This sequence harbors 28 insertions, or 83–85 per mB of target. Promoter set II comprises 324–340 kB of promoter sequence, and harbors 24 insertions or 70–74 per mB. Promoter set III comprises 330–343 kB of promoter sequence, and harbors 19 insertions or 55–57 per mB. These frequencies do not differ significantly (3-way G-test, p = 0.40, 0.37, and 0.39 depending on whether the target size correction is conservative, semi-conservative, or liberal, respectively.) By contrast, the promoters of *Hsp70* comprise 40–48 kB of promoter sequence and harbor 19 insertions, or 395–476 per mB. As noted, not every promoter was recognizable in all 12 species. When the analysis is repeated for promoters recognizable in only 5 species (the *D. melanogaster* subgroup species) or only 8 species (all but *D. willistoni*, *D. mojavensis*, *D. virilis* and *D. grimshawi*), the result is essentially the same except that statistical significance is less (Table S3).

The pattern for natural populations of *D. melanogaster* [2] may differ from that in the present study for a second reason: the former study summed insertions into multiple alleles (minimally 1 per each of 48 populations), whereas the present study examines far fewer alleles (minimally 1 per each of 12 species). Accordingly, we have compared the distributions of TE insertions per allele for the 3 promoter sets of [2]; these differ (Kruskal-Wallis test, p<0.0001). Excluding *D. melanogaster* (because the sequenced strain was selected for the absence of one TE family) and comparing the 11 remaining sequenced species in the same way but for the promoter sets of the present study, we find that the distributions do not differ (p = 0.52).

Alternatively, the distributions of TE insertions in the genesets of the present study can be compared in phylogenetic context. The phylogenetic trees in Fig. 2 present 17 tips plus nodes (i.e., branchpoints of two clades) at the 12-species level, 13 at the 8-species level, and 8 at the 5-species level. Older nodes are excluded because no TE insertions are detectable at them, presumably because of degeneration over time. For all possible pairwise comparisons of the frequency distributions of TEs at tips plus nodes, the promoter sets do not differ (Kolmogorov-Smirnov two-sample test, 0.31>p>0.06), even without a Bonferroni correction.

### Position of TE Insertions in Promoter Regions

**Figure 4:**
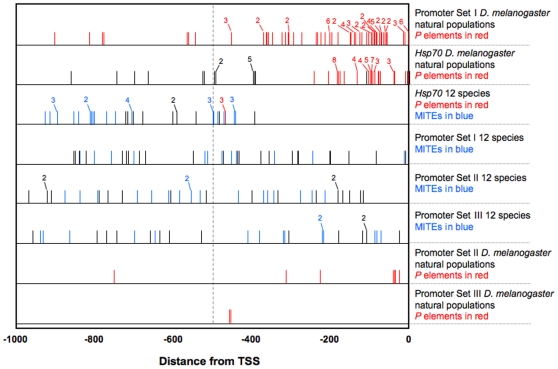
Insertion sites of TEs in three single-copy promoter sets and *Hsp70* promoters in the 12 sequenced genomes, and in similar promoters of natural populations of *D. melanogaster*. Data for *D. melanogaster* natural populations was taken from [2]. Due to shared insertions in all genesets, and gene duplication and the spatial arrangement of *Hsp70* copies, some individual TE insertions occur in more than one promoter. Distance to TSS of shared insertions is shown for all promoters in which the insertion is found.

The average distance between TE insertions and the TSS did not vary significantly among promoter sets (mean Promoter set I: 479 bp; Promoter set II: 515 bp; Promoter set III: 451 bp; Mann-Whitney U-test, I vs. II: p = 0.704, I vs. III: p = 0.624, II vs. III: p = 0.337). Insertions into promoters of *Hsp70* are further from the TSS than are insertions into the three single-copy promoter sets (mean *Hsp70*: 658 bp), although no pairwise comparison is significant after Bonferroni correction (Mann-Whitney U-test, *Hsp70* vs. I: p = 0.0016; *Hsp70* vs. II: p = 0.016; *Hsp70* vs. III: p = 0.0045). These patterns contrast to those for *D. melanogaster* natural populations [2]. We calculated distances to the TSS for insertions listed in [2] and compared them to distances calculated in the present study. The distances differ most markedly for *Hsp70* (Mann-Whitney U-test, p<0.0001), into which most TEs in *D. melanogaster* natural populations inserted within 400 bp of the TSS [2]. No TEs in the 12 species' genomes inserted in this interval. For single copy heat-shock genes, the distances are more similar in the two studies (Figure 4), but in *D. melanogaster* natural populations are nearer the TSS ( p<0.0001).

For the three single-copy promoter sets in the present study, TEs are inserted randomly across the entire region from the TSS to 1 kb upstream, whereas insertions into both *D. melanogaster* natural population heat-shock promoters and *Hsp70* promoters of the 12 species are not (Figure 4). The latter sets deviate from a Poisson distribution (12 species *hsp70*: chi-square, p<0.0001, *Hsp70* in *D. melanogaster* natural populations: p<0.0001; Set I promoters in *D. melanogaster* natural populations, p<0.0001), whereas those inserting into the genesets for the 12 species do not (Promoter set I: p = 0.743, Promoter set II: p = 0.279, Promoter set III: p = 0.272).

### Extent of TE Sequence in Proximal Promoters with TE Insertions

The functional consequences of TE insertions may depend on their size. Lengths of TE sequence were similar in the three promoter sets (I: 158 bp mean, II: 175 bp, III: 97 bp). Length estimates intentionally excluded portions of TEs extending outside the 1000 bp window. Because promoter sets did not differ in TE insertion position, however, any bias due to this exclusion should apply equally to all promoter sets. Insertion sizes did not differ among promoter sets, even without Bonferroni correction (Mann-Whitney U-test, I vs. II: p = 0.142; I vs. III:p = 0.810; II vs. III: p = 0.066). The mean length of insertions in *Hsp70* promoters (177 bp) only differed significantly from Promoter set III after Bonferroni correction (Mann-Whitney U-test, I v. *Hsp70*: p = 0.014, II v *Hsp70*: p = 0.271, III v. *Hsp70*: p = 0.0003).

Sequence conservation of TE insertions can elucidate the evolutionary forces acting on these elements. Few insertions recovered in this study are full-length, but instead are fragments. We compared these fragments with their best-hit consensus or species-specific centroid sequence [18]. Although the average percent of consensus TE length varies among the three single copy promoter sets and *Hsp70* promoters (average % of consensus: Promoter set I = 17.8%, II = 24.9%, III = 11.8%, *Hsp70* = 19.5%), no pairwise comparisons among genesets on this metric are significant after Bonferroni correction (Mann-Whitney U-test, I vs. II: p = 0.089, I vs. III: p = 0.818, II vs. III: p = 0.013, I vs. *Hsp70*: p = 0.093, II vs. *Hsp70*: p = 0.465, III vs. *Hsp70*: p = 0.004).

### TE Composition

The majority of TEs detected in this study are *INE-1* type [19], which are small, non-autonomous miniature inverted-repeat elements (MITEs), totaling 26 of 71 (37%) insertions in the three single-copy promoter sets, and 11 of 19 insertions in the *Hsp70* promoters (Table S2). Of the remainder, 25 are novel repetitive elements [18], 5 are LTR retrotransposons, 2 are *Penelope* retrotransposons, and 16 are DNA transposons.

## Discussion

First, as a caveat, we re-emphasize that the promoter sets chosen in the previous study of natural populations of *D. melanogaster* [2] and the present study of 12 sequenced genomes are similar but not identical. The dissimilarity arises because orthologues of the promoters examined in the prior work are not unambiguously recognizable in enough of the 12 species genomes for a powerful comparison of inserted TEs. The rationale for the selection of included promoters is in Table S1.

The whole-genome sequencing of *Drosophila melanogaster* [20] provided a first glimpse at the “transposome” of a complex eukaryote [21]. First glimpses may not illuminate general patterns, however, because they reflect the distinctive features of species and strains chosen for sequencing. Thus, our initial finding of numerous TEs in proximal promoter regions of heat-shock genes of natural populations of *D. melanogaster* [2] was potentially idiosyncratic because it largely involved a TE (the *P* element) absent in both the strain chosen for sequencing and in some other *Drosophila* species [20], [22], [23]. Is this finding unique to natural populations of *D. melanogaster*, or more general? Whole-genome sequences now available may answer this question.

The genome sequences for the 12 *Drosophila* species still yield the ascertainment bias inherent in the choice of strains for sequencing - a bias soon to diminish as resequencing projects progress [24]. Nonetheless, they clearly indicate that the preponderance of *P* elements in proximal promoter regions of heat-shock genes is not universal in the genus *Drosophila*. First, in contrast to the natural populations of *D. melanogaster* screened by [2], the majority of the insertions recognized in the present study are MITEs. MITEs also abound in plants, in which they frequently insert in gene-rich regions and may figure prominently in the evolution of gene expression [25]–[29]. Indeed, these parallels between plants and *Drosophila* suggest that MITEs may play similar roles in diverse eukaryotes.

TE insertions into promoters in natural *D. melanogaster* populations differ from the aggregate pattern from the 12 single species genomes in a second key way. In the former, insertions are more numerous in promoters of single-copy heat-shock genes, where they cluster near the TSS, than in equally-sized control promoter sets. By contrast, for the 12 genomes as a group, insertions into promoters of single-copy heat-shock genes are not more numerous than into equally-sized control promoter sets, and are randomly distributed throughout the 1000 bp window screened.

The first of several explanations for the lack of concordance in results between *D. melanogaster* natural populations and the 12 species whose genomes were sequenced is that the Universal Fast Walking (UFW) technique of the former work failed to detect TEs that whole-genome sequencing revealed. Because UFW incorporates PCR, which can favor shorter amplicons, it is theoretically possible that UFW might be biased towards discovering only TEs close to the TSS. We disfavor this possibility for several reasons: (1) An actual bias has not been documented to date for UFW (K. Myrick and W. Gelbart, pers. com.), nor is one evident in other published uses of the technique. (2) Walser et al. [2] screened a full kB upstream of the TSS and reported more (i.e., not fewer) TEs than revealed through whole-genome sequencing.

A second explanation for the comparative rarity of TEs in Promoter set I of the present study is that extensive rescreening in the former (n = 48 populations) reveals rare TE insertion alleles not evident in the smaller sample from single genomic sequences. As the individual sequenced genomes sample only a limited proportion of the variation within-species, some mutations, especially low-frequency mutations such as some TE insertions, are unlikely to be discovered. We attempted to address this possibility in two ways. First, we reanalyzed the data of [2] by performing 20 random draws of 12 populations each. In every case, TE insertions into Promoter set I significantly outnumbered TE insertions into the other single-copy promoter sets. Presumably, if a similar pattern occurred in the sequenced species' genomes, a sample size of 12 would be sufficient to detect it. Second, we directly compared the distributions of TE insertions per allele for the 3 promoter sets of [2] and per genome for the 3 promoter sets of the 11 sequenced species. These comparisons recapitulated outcomes of comparisons in which TEs are simply aggregated for each Promoter set (see RESULTS).

A third explanation is that *D. melanogaster* is a conspicuous outlier in its number and dynamics of TEs in general. Interspecific comparisons of overall repetitive sequence content and TE composition [1], [18] and of *INE-1* element dynamics [19] in the 12 genomes do not support this explanation. The plurality of MITEs in the present study is consistent with their high copy number in all *Drosophila* genomes [19]. MITEs are as numerous in the 3 promoter sets (25–26 MITEs per Mb using conservative and liberal target size corrections for the 3 promoter sets) as in average noncoding sequence of the 12 sequenced genomes (2.7–60.0 per Mb of noncoding sequence [1], [19]). Using as a basis the number of annotated repeat insertions in the 12 species genomes (Caspi, unpublished) obtained through a comparative alignment method [30], we estimated the total number of TE insertions per Mb in the sequenced genomes. These estimates indicate that TEs average 129–330 per Mb of average non-coding sequence vs. 69–72 for the 3 single-copy promoter sets (depending on whether the liberal or conservative correction for target size is applied) and 395–476 for the *Hsp70* promoter set. Both comparisons are inexact, however, due to methodological differences and because only the present study differentiates between promoter vs. non-promoter noncoding sequence. Vertical acquisition of TE insertions (i.e., inheritance of a TE acquired prior to species divergence) in *D. melanogaster*, which the present study implicates, is both evident in other *Drosophila* species and is reported elsewhere [31]. Finally, the promoter sets chosen for study may reflect some inadvertent bias unrelated to the main hypothesis, either in general or in *D. melanogaster*. For example, if heat-shock genes are more numerous due to gene duplication in *D. melanogaster*, the target for and/or tolerance of TE insertion would be larger in *D. melanogaster* populations than in other species. With respect to this example, the total number of orthologues for the *D. melanogaster* single-copy heat-shock promoters is no less in the other 11 species than in *D. melanogaster* (Table S4). An additional peculiarity is that the proportions of TEs other than MITES in the 3 genesets are not consistent with that found in the genomes as a whole [1], although sample sizes are small. LTR retrotransposons are underrepresented in the present study, and novel elements are overrepresented.

As the Introduction reviews in detail, the natural populations of *D. melanogaster* and some of the 12 species differ in ways relevant to the main hypothesis, including:

- activity of a TE (the *P* element) with a distinctive insertional mechanism and corresponding insertion site preference. This explanation receives some (but incomplete) support because *P* elements have not been detected in many of the 12 species [22], [23]. *P* elements occur in *D. willistoni*, however, which appears similar to the other 11 species in overall TE distribution in this study. *D. willistoni* also has the second highest level of repeat coverage of the sequenced genomes [1]. Further screening of single copy heat-shock genes in multiple *D. willistoni* populations has revealed no *P* element insertions (unpublished data). The *D. willistoni* situation shows that the presence of *P* elements, even within a genome where TEs appear to have been highly active, may not in itself be sufficient to yield the TE distribution characteristic of the *D. melanogaster* natural populations.
- recency of the horizontal transmission of *P* elements to *D. melanogaster*, which could contribute to the high number of insertions in *D. melanogaster* heat-shock promoters. Unless some mechanism (e.g., positive selection, duplication, gene conversion) preserves them, TEs may be subject to degeneration once inserted and immobilized, and may become unrecognizable. [2] reported substantial degeneration of *P* elements in the natural populations of *D. melanogaster*. In our sample, few insertions are complete TEs and most retain on average ∼20% of their canonical sequence. For TEs shared among species, times of lineage divergence place upper bounds on the insertion times: no insertion older than 12 MYA is recognizable. Recent invasions will be more recognizable, all else equal. The *P* element is believed to have invaded *D. melanogaster* within the last 100 years [32]. The recency of *P* elements' invasion thus may make their presence conspicuous in *D. melanogaster* populations. However, recent activity alone cannot account for the restriction of the pattern to *D. melanogaster*. Other TEs, such as MITEs, have been recently active in many of the species [19], and are commonly found in promoter regions in this study. Yet, they are not in excess in single copy heat-shock promoters. In addition, recent activity of the *P* element has also been documented in *D. willistoni* [33], yet no *P* elements were found in any *D. willistoni* promoter region.
- habits and habitats that predispose *D. melanogaster* to heat shock in nature, accounting for distinctive patterns of heat-shock gene expression. *D. melanogaster* is cosmopolitan, encountering diverse macroclimates, and its non-adult stages occur in microhabitats (necrotic fruit) that are prone to thermal stress [17]. These features may select for retention of TE insertions into heat-shock genes, depending on the TEs' impact on gene expression. A weakness of this candidate explanation, however, is that others of the 12 species (e.g., *D. simulans*, *D. virilis*) share many of these same attributes [34].

Thus, none of these possible distinctions in isolation provides a definitive explanation for the restriction of the excess of TE insertions in single-copy heat-shock promoter regions to natural populations of *D. melanogaster*. Possibly the particular combination of these characteristics produces the pattern. Nonetheless, a robust explanation is currently lacking.

The multi-copy promoter regions of *Hsp70* are an exception to the findings for single copy heat-shock promoter regions. First, in both the natural populations of *D. melanogaster* and in the aggregate sequenced genomes of the 12 species, *Hsp70* promoters harbor more TE insertions per promoter than any of the single-copy promoter sets. Second, the positions of natural and experimental TE insertions into *Hsp70* promoters in the natural populations of *D. melanogaster* are nearly exclusive of the positions of insertions into *Hsp70* in the sequenced genomes of the 12 species (Figure 4). Third, for the *D. melanogaster* natural populations, insertions into *Hsp70* promoter regions include TEs other than *P* elements [6], [35].

In principle, *Hsp70*-specific hotspots could account for the numerous TE insertions into this gene's promoters. Indeed, the *Hsp70* promoter presents a confluence of DNAse hypersensitivity, decondensed chromatin, nucleosome-free regions, and flanking GAGA elements [and, for *P* elements, preferred insertion sequence] [5], [36]. Although other heat-shock promoters have not been investigated as thoroughly, they share these features [3], [37], which are thus not specific to *Hsp70*. Relative to single-copy heat-shock genes *Hsp70* is more massively expressed, is perhaps more sensitive to induction, and obviously is multicopy. Insertion of TEs into promoters of single-copy heat-shock genes can severely reduce or eliminate expression of the affected gene, whereas insertion into a single *Hsp70* promoter can leave many other copies unaffected, leading to reduced negative selection against insertions. Additionally, insertion of a TE into a single *Hsp70* promoter creates allelic variation that is apparently responsive to positive selection [12], [15].

Why the positions of these insertions into *Hsp70* promoters (Fig. 3) differ so dramatically between the *D. melanogaster* natural populations and the 12 species' sequenced genomes is inexplicable at present. As noted above, the prevalence of TEs near the TSS in *D. melanogaster* may reflect a bias in the UFW technique. As also noted above, such a bias is yet to be documented. Additionally, as the sequenced genomes are not prone to this bias yet contain no insertions in *Hsp70* promoters within approximately 400 bp of the TSS, the insertion patterns still differ between *Hsp70* promoters in the natural populations and in the sequenced genomes even if a bias exists.

The *Hsp70* proximal promoter sequences, and presumably architectures, are grossly similar throughout the 12 species (unpublished data). At least in *D. melanogaster*, negative impact on *Hsp70* expression declines with distance of the TE insertion from the TSS [38]. By implication, therefore, large insertion-mediated reductions in a single *Hsp70* copy are well-tolerated in natural populations of *D. melanogaster*, but not in strains/species whose genomes have been sequenced. No clearcut biological explanation for this implication presents itself.

In summary, the whole-genome sequencing of 12 species of *Drosophila* has elucidated both functional and evolutionary patterns of fundamental importance. In the present study, the genomic sequences clarify that both *Drosophila melanogaster* as a species and *Hsp70* as a gene are distinctive in terms of their interaction with transposable elements. Nonetheless, the biological underpinnings of this distinctiveness remain elusive.

## Materials and Methods

### Geneset Definition

Promoter sets for analysis follow those of [2]. Promoter set I includes heat-shock genes, in whose promoters *P* elements are abundant in *D. melanogaster*. Promoter set II excludes heat-shock genes but includes genes sharing one or more features with heat-shock genes, such as constitutively decondensed chromatin, lack of nucleosomes and presence of DNase hypersensitive sites in the proximal promoter region [3], regulation via polymerase II pausing, and high transcription in germline cells. Promoter sets I and II are expected to have similarly high exposure to transposable element insertion. Promoter set III members, by contrast, exhibit temporally limited transcription with low germline transcription, characteristics that should minimize transposon insertions. As will be described, the unambiguous identification of orthologs and/or their corresponding transcription start sites for all *D. melanogaster* promoters used in [2] was impossible. Table S1 indicates genes from [2] in which some or no orthologous transcription start sites could be identified. To maintain the power of the comparison, we added genes to each set until we achieved the following arbitrarily-chosen sample size: 12 in each set identifiable in all 12 species, 15 identifiable in only 8 species (*D. melanogaster* subgroup +*D. ananassae*, *D. pseudoobscura* and *D. persimilis*), and 12 identifiable in only the 5 *D. melanogaster* subgroup species. These promoters were included before scrutiny of their TEs.

We added to the original promoter sets through the incorporation of genes not included in [2], but with characteristics warranting inclusion [39]–[41]. Details of all characteristics used to classify genes are listed in Table S1, and column K shows all characteristics pertaining to a given gene.

### Evaluation of Transcription Start Sites (TSS) in *D. melanogaster*

The search for TEs upstream of the TSS is contingent upon the unambiguous identification and localization of the TSS, which we describe here. All TSS from a total of 107 genes were evaluated first in *D. melanogaster*. The 107 genes (excluding *Hsp70*: see below) across the three genesets included 38 genes with multiple transcripts in *D. melanogaster*, for a total of 148 transcripts in the 107 genes. Twenty-seven of these multiple-transcript genes have transcripts that differ in putative transcription start sites (TSS) and thus presumably also differ in their associated promoter regions (Table S1). If a given gene contained more than one putative TSS and these start sites were separated by 500 bp or more (∼50% of data analyzed per gene per species), we retained both TSS and associated upstream promoter region for further analysis, for a total of 117 TSS/promoter regions considered (39 per geneset).

To determine the position of TSS for genes in *D. melanogaster*, we compared annotated transcripts and 5′ UTR to existing experimental data. First, 42 genes have an experimentally determined transcription start site (TSS) included in the Drosophila Core Promoter Database [42], or the Eukaryotic Promoter Database [43]. An additional 60 TSS without DCPD or EPD entries had associated full-length or full-insert cDNA [44], [45] catalogued in Flybase. Although no attempt was made to ensure that the cDNA represented the entire transcript, these cDNA provide some experimental support. The remaining 15 TSS were defined on the basis of an annotated UTR only.

From Flybase we extracted the coding region and sequence upstream from the start codon for each gene in *D. melanogaster*. Genomic sequence, experimentally determined promoter sequences with defined TSS, full-insert and full-length cDNA from Flybase, and annotated 5′ UTR or transcripts were aligned as available for each gene with CLUSTAL in Bioedit 7.0.5.3 [46] and manually adjusted when necessary.

For the 42 experimentally determined TSS from the DCPD and EPD databases, only three agreed completely with the annotated 5′ UTR on TSS location. Only three full-insert or full-length cDNA catalogued in Flybase had the same TSS as the promoter database entry. However, in general the deviation from the experimentally confirmed promoter database TSS was small, averaging 20.91 bp (n = 37; SD = 32.6) for full-insert or full-length cDNA and 44.6 bp (n = 41; SD = 64.1) for annotated UTR. When the EPD/DCPD TSS was at odds with the Flybase annotated 5′ UTR entry or catalogued cDNA, we used the EPD/DCPD TSS.

Of the 60 additional TSS that were supported by a cDNA in Flybase, for 43 the 5′ end coincided with the annotated TSS position in Flybase. For those that did not coincide the average deviation was only 31.35 bp (N = 98; SD = 69.5). Whenever a full-length or full-insert cDNA was available, we based the TSS on cDNA. As will be seen, however, minor deviations in placement of the TSS would have little impact on the results.

### *Hsp70* TSS Determination

Coding and upstream sequence were obtained at Flybase for the six copies of *Hsp70* in the *D. melanogaster* sequenced strain. A single EPD entry for *Hsp70* was aligned to the sequences along with 5 full-insert or full-length cDNA. The five full-insert and full-length cDNA vary in putative TSS. First, of the two full-insert cDNA for *Hsp70Bc*, one (BT011541) is substantially longer than the other, and is highly divergent at the 5′ end, both from the other cDNA and from the genomic sequence. This sequence was excluded from the analysis. Of the remaining four cDNA, the TSS is identical in three, and 7 bp upstream in the fourth. Five of the six annotated UTRs or mRNAs indicate TSSs occurring within a narrow window of 16 bp. By contrast, the UTR annotated for *Hsp70Aa* is far longer, terminating 218 bp 5′ to the known TATA-box promoter [47], [48], and is hence assumed to be misannotated. All other cDNA and annotated UTR have TSS consistent with the position of the conserved TATA-box.

### Orthology Determination and Sequence Extraction

To obtain orthologues of *D. melanogaster* genes from *Drosophila* species genomes, BLASTn and tBLASTn were performed at the Flybase website [49] with *D. melanogaster* coding sequences, gene region sequences, or protein sequences. BLAST was performed to *D. melanogaster* release 5.10, *D. pseudoobscura* release 2.3, *D. virilis* release 1.2, and release 1.3 for all other species. Of the 107 genes across three genesets examined in this study, excluding *Hsp70* (which is known to be multicopy [50]), 70 have 1∶1 orthologues in all other sequenced genome species of *Drosophila* [1].

Of the remaining genes, we carefully screened using BLAST, Gbrowse orthology calls and reciprocal BLAST to the annotated *D. melanogaster* genome to determine the number of copies in each species. Only genes with predicted coding sequences in all species considered were included. Fourteen genes appeared to have more than one copy in at least one additional species at the phylogenetic depth at which they were analyzed (Table S1). We verified that duplicate copies had complete open reading frames by referring to predicted coding sequences in Flybase or Genscan [51]. If a full-length or near full-length coding sequence was not present, the copy was discarded as a pseudogene; otherwise it was retained for further analysis. Twenty duplicates in the fourteen genes met this criterion and were retained for assessment of TSS conservation (see below).

In each species, we initially extracted 3000 bp upstream of each identified gene copy. Alignments of gene regions were performed with CLUSTAL in Bioedit [46] and more upstream sequence was acquired when necessary to account for the presence of length variation among species, particularly within 5′ UTR introns.

### *Hsp70* Data Collection and Alignment

We used BLASTn with the coding sequence of *D. melanogaster Hsp70Aa* as a query to each of the *melanogaster* subgroup genomes. All hits with an e-value of 0.0 were further examined to determine whether they constituted a putative copy of *Hsp70*, using a combination of Gbrowse orthology calls, synteny and relative position, and reciprocal BLAST to *D. melanogaster*. We included all verified copies found in each species in alignment. We identified four putative copies in the genomes of *D. simulans*, *D sechellia* and *D. yakuba* and six in *D. erecta*. While coding regions were complete for all *D. erecta* copies, gaps, often surrounded by variable length stretches of divergent sequence, interrupted the coding regions of 2 *Hsp70* copies each from *D. simulans* and *D. sechellia*. All *D. yakuba* copies had gaps in the coding region, and two had divergent sequence surrounding the gap. Previous analysis had identified four copies in *D. simulans*, *D. sechellia* and *D. yakuba*, while copy number of *Hsp70* in *D. erecta* was not assessed [50]. We therefore assume that the coding region gaps in *D. simulans, D sechellia* and *D. yakuba* represent sequencing and assembly errors, and all are considered functional copies and included in further analysis, with one exception. The coding region gap in one of the copies of *Hsp70* from *D. simulans* encompasses the 5′ end of the coding region and no alignment with the other *Hsp70*, including those from *D. simulans*, is found upstream of this gap. We are thus lacking the proximal promoter including the TATA box and putative TSS and cannot define the region to be analyzed for this copy, which is therefore excluded.

We followed the same procedure as outlined for the species of the *melanogaster* subgroup to collect putative orthologues of *Hsp70* in the other seven *Drosophila* species for which genome data is available. In all species, multiple copies were recovered (*D. ananassae*: 5, *D. pseudoobscura*: 2, *D. persimilis*: 2, *D. willistoni*: 3, *D. grimshawi*: 3, *D. virilis*: 6, *D. mojavensis*: 6). In species where copy number of *Hsp70* was previously examined [52], [53] our findings were in agreement, with the exception of an additional copy found in *D. willistoni*.

### Conservation of *D. melanogaster* TSS Across Species

For each distinct TSS in *D. melanogaster*, we examined conservation across species using the UCSC genome browser and threaded-blockset alignments produced by Multiz via the PromAn website [54]. As in the UCSC genome alignments, this approach uses a BLASTz search to first identify homologous regions before aligning conserved blocks, and was shown to be more effective than other alignment algorithms at aligning highly diverged sequence, such as may be found in non-coding regions in between-species comparisons [55]. We assume that conservation of sequence in the region encompassing the TSS implies functional conservation although this is not always necessarily the case [56].

Levels of putative TSS conservation varied among genes. Of the 117 TSS identified in *D. melanogaster*, 36 had a conserved block in all 12 species that included the *D. melanogaster* TSS. To keep species composition approximately constant across genesets, average out species-specific effects, discover general trends, and broaden sampling, we also analyzed 45 additional TSS that were conserved across only the eight species of the *D. melanogaster* and *D. obscura* groups, and 36 that were conserved only in the five species of the *D. melanogaster* subgroup. For *Hsp70*, all putative copies were conserved at the TSS and through the proximal promoter, and included a conserved TATA box motif [47]. If data were missing for a species in the region encompassing the TSS, this was counted as lack of conservation. In some cases, only a single paralogue was alignable at the TSS, and this copy was retained for further analysis. Not including *Hsp70*, 8 paralogues in 6 genes were conserved at the TSS. These duplicate copies are not equally distributed among genesets (5 in geneset I, 3 in geneset III) but only account for a small additional amount of analyzed sequence (1-2% of total) per geneset and hence were included in analysis.

Alignments were trimmed to include the region 50 bp downstream from the TSS and 1000 bp upstream, a region that should encompass the majority of proximal promoter regulatory elements associated with a given gene [57]. All alignments were converted to BLAST databases using the formatdb command in BLAST v. 2.2.16.

### Discovery of Transposable Elements

Databases produced from alignments were submitted to BLASTn searches using (1) Repbase *Drosophila* transposable elements [58], (2) the curated *D. melanogaster* (version 9.4.1) set maintained at the Berkeley Drosophila Genome Project and (3) the non-redundant set of *de novo* PILER repetitive element predictions for the 12 *Drosophila* species genomes [18]. Hits at 1e^-05^ were considered positive. In the case of multiple hits by related TE to the same location, only the highest scoring hit was retained. Results of searches with the three databases were manually compared and reconciled. Genome coordinates for TE sequence within all sampled promoters are included in Table S5.

### Statistical Tests

To test for significance in pairwise and three-way comparisons in the observed number of distinct transposable element insertions in genesets versus an expected number based on a null hypothesis of equal probability of insertion into any individual promoter region included in the analysis, we used G-tests with the degrees of freedom scaled to the number of categories in a given comparison (Table S3). We used the Kruskal-Wallis test to compare the distribution of the number of TE insertions per line or species among genesets. A Kolmogorov-Smirnov two-sample test was used to determine if the phylogenetic distribution of insertions varied among genesets at each level of species sampling (Table S6).

We used non-parametric Mann-Whitney U-tests to determine if distance from the TSS, extent of TE sequence in promoter regions or percent element length relative to consensus or centroid length was different among genesets. As all sets of values compared had sufficient sample sizes, probability in the Mann-Whitney U-tests was assessed with the z statistic (Table S6).

We tested whether the distance of insertions from the TSS fit a Poisson distribution in the sampled interval by comparing observed and expected occurrences of downstream TE boundaries in 50 bp bins across the interval. Significance was assessed with a chi-square test with degrees of freedom dependent on the maximum number of TE insertions within an interval (Table S6).

The experiment-wide false positive rate was conservatively controlled using a standard Bonferroni correction for multiple tests.

## Supporting Information

Table S1. Promoter regions and transcription start sites (TSS) sampled. The basis of TSS definition in *D. melanogaster* as defined in the text is listed in Column G. All characteristics pertaining to the inclusion of a promoter in a given promoter set are in Column K, and the key to these characteristics is below the table. The small number of analyzed duplicate promoters are identified in Column J.
(0.04 MB XLS)

Table S2 TE insertions in promoters of 12 species genomes. Each distinct insertion is listed with the promoter it was found, and all species in which it was identified. E-value refers to the best BLASTn hit. Element length indicates the amount of TE sequence in the defined promoter region. The identity of the TE is listed along with TE class (retrotranposon = I, DNA transposon = II).
(0.04 MB XLS)

Table S3 TE insertions per bp in each promoter set and 3-way G- test results at three levels of species sampling. Liberal, semi-conservative and conservative refer to method of estimating promoter set target size.
(0.02 MB XLS)

Table S4 Number of orthologues of *D. melanogaster* heat-shock genes in each *Drosophila* species
(0.02 MB XLS)

Table S5 Genomic location of TE insertion sequence in *Drosophila* promoters. For each insertion, in all promoters in which it is found, the genomic coordinates of both the promoter region and the TE sequence contained within it are tabled. Coordinates are from genome releases as detailed in the text. The first sheet contains insertions in promoters from all single copy genes, while insertions from the multicopy *hsp70* genes are listed in the second sheet.
(0.06 MB XLS)

Table S6 Values of test statistics, degrees of freedom where applicable, and significance level for statistical tests.
(0.02 MB XLS)
